# Cinnamic Aldehyde Inhibits Lipopolysaccharide-Induced Chondrocyte Inflammation and Reduces Cartilage Degeneration by Blocking the Nuclear Factor-Kappa B Signaling Pathway

**DOI:** 10.3389/fphar.2020.00949

**Published:** 2020-08-05

**Authors:** Pu Chen, Anmin Ruan, Jun Zhou, Liuwei Huang, Xiaozhe Zhang, Yufeng Ma, QingFu Wang

**Affiliations:** ^1^ Third Affiliated Hospital, Beijing University of Chinese Medicine, Beijing, China; ^2^ Department of Nephrology, Southern Medical University, Guangzhou, China; ^3^ Department of Orthopedics, Beijing University of Chinese Medicine Third Affiliated Hospital, Beijing, China

**Keywords:** cinnamic aldehyde, knee osteoarthritis, chondrocyte inflammation, chondrocyte degeneration, NF-kB signaling pathway

## Abstract

Osteoarthritis (OA), as one of the top 10 causes of physical disability, is characterized by inflammation of the synovial membrane and progressive destruction of the articular cartilage. Cinnamic aldehyde (CA), an α,β-unsaturated aldehyde extracted from the traditional Chinese herbal medicine cinnamon (*Cinnamomum verum* J.Presl), has been reported to have anti-inflammatory, antioxidant, and anticancer properties. However, the anti-inflammatory effect of CA on OA remains unclear. The purpose of the present study was to investigate the effects of CA on inflammation, and cartilage degeneration in OA. A CCK-8 assay was performed to assess the potential toxicity of CA on cultured human OA chondrocytes. Following treatment with lipopolysaccharide (LPS) and CA, the expression of proinflammatory cytokines, including interleukin (IL)-1β, IL-6, and tumor necrosis factor-alfa (TNF-α), was evaluated using quantitative real-time polymerase chain reaction (RT-qPCR) analysis, enzyme-linked immunosorbent assay, and Western blotting (WB). The production of matrix metalloproteinase-13 (MMP-13) and a disintegrin and metalloproteinase with thrombospondin motifs 5 (ADAMTS-5) was also examined using RT-qPCR and WB. Furthermore, to investigate the potential anti-inflammatory mechanism of CA, biomarkers of the nuclear factor kappa-light-chain-enhancer of activated B cells (NF-κB) pathway (p65, IKB-α) were detected using WB. The results demonstrated that CA significantly inhibited the expressions of IL-1β, IL-6, TNF-α, MMP-13, and ADAMTS-5 in LPS-induced OA chondrocytes. CA dramatically suppressed LPS-stimulated NF-κB activation. Collectively, these results suggest that CA treatment may effectively prevent OA.

## Introduction

Knee osteoarthritis (KOA), one of the most common degenerative musculoskeletal diseases involving chronic pain and dysfunction (Felson et al., 2000), is characterized by inflammation of the synovial membrane and progressive destruction of the articular cartilage (Berenbaum, 2013; Malemud, 2015). KOA has been reported to be one of the top 10 causes of physical disability (Neogi, 2013); however, no treatment methods developed to date have been able to reverse or alter the progression of KOA (Chen et al., 2019). Although the pathogenesis of KOA remains unclear, inflammation is widely regarded to be an extremely important factor (Kapoor et al., 2011; Berenbaum, 2013; Yang et al., 2016). Cartilage damage has been reported to induce synovial inflammation (Scotece et al., 2018), and chondrocyte‐mediated inflammatory responses play a key role in the development and progression of osteoarthritis (OA) (Konttinen et al., 2012). Therefore, effective inhibition of chondrocyte inflammation may play a role in reversing or altering the progression of OA.

Many signaling pathways play a crucial role in the development and progression of OA. The nuclear factor kappa-light-chain-enhancer of activated B cells (NF-κB) signaling pathway is widely distributed in various parts of the body, mediating immune responses, inflammation, and stress responses (Lee and Burckart, 1998; Sun, 2017; Zhang et al., 2017). In general, NF-κB is in an inactive state because its dimer can interact with an inhibitory protein, inhibitory protein of NF-κB (IKB), which binds to a trimer complex under non-stimulated conditions. After receiving stimulus, IKB kinase is activated by phosphorylating the IKB protein, thereby dissociating from the trimer, and then activating the NF-κB signaling pathway. Activated NF-κB can induce excessive expressions of interleukin (IL)-1β and matrix metalloproteinases (MMPs), which play key roles in inflammation and extracellular matrix (ECM) degradation (Santangelo et al., 2012; Kepczynska et al., 2017). In addition, IL-1β can induce the synthesis of inducible nitric oxide synthase (iNOS) and stimulate the production of nitric oxide (NO), cyclooxygenase-2 (COX-2), prostaglandin E2 (PGE2), and tumor necrosis factor- alpha (TNF-α) (Goldring and Goldring, 2004; Bianchi et al., 2005; Wang et al., 2011; Fioravanti et al., 2012), which is associated with OA progression. Furthermore, IL-1β can also stimulate the release of MMPs and a disintegrin and MMP with thrombospondin motifs (ADAMTS) (Santangelo et al., 2012); MMPs and ADAMTS can significantly promote the degradation of ECM, which further aggravates the progression of OA.

**Figure 1:**
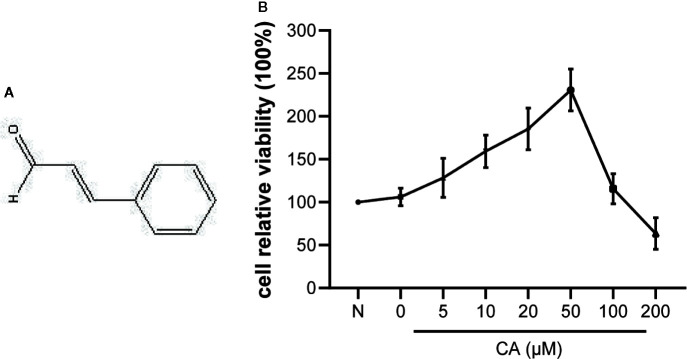
Effect of cinnamic aldehyde (CA) on the viability of human osteoarthritis (OA) chondrocytes. **(A)** The chemical structure of CA. **(B)** Human OA chondrocytes were pretreated with different concentrations of CA (0, 5, 10, 20, 50,100, and 200 μM) for 24 h and evaluated using the CCK-8 assay.

Cinnamic aldehyde (CA) is an α,β-unsaturated aldehyde extracted from the traditional Chinese herbal medicine cinnamon (*Cinnamomum verum* J.Presl). The chemical structure of CA is shown in **Figure 1A**. Several studies have suggested that cinnamon extract has anti-inflammatory, antioxidant, antidiabetic, antipyretic, antibacterial, and anticancer properties (Matan et al., 2006; Kwon et al., 2010; Sung et al., 2011; Wang et al., 2015; Buglak et al., 2018). However, the anti-inflammatory effect of CA on OA remains unknown. Therefore, the aim of the present study was to investigate the anti-inflammatory effect and underlying mechanism of CA in chondrocyte inflammation and degeneration.

## Methods

### Articular Chondrocyte Isolation and Culture

The present study adhered to the principles of the Declaration of Helsinki, and the protocol was approved by the ethics committee of the Third Affiliated Hospital of Beijing University of Chinese Medicine (Beijing, China). All human clinical samples were obtained from patients who underwent total knee replacement surgery at the Third Affiliated Hospital of Beijing University of Chinese Medicine. Deidentified, discarded human cartilage samples were used for this study. Articular cartilage samples were collected from 6 patients who fulfilled the American College of Rheumatology classification criteria (Palmieri et al., 2010) for the diagnosis of OA (2 men and 4 women; 62 to 73 years of age). Briefly, cartilage samples were washed in phosphate-buffered saline (PBS), which included an antibiotic mixture (penicillin/streptomycin, Invitrogen/Thermo-Fisher Scientific, Waltham, MA, USA) 3 times and then diced into 1×1×1 mm^3^ pieces. These joint cartilage pieces were digested with 0.25% trypsin-EDTA solution (Solarbio, China) for 40 min and then overnight at 37°C in 0.2% type II collagenase and centrifuged at 176 g for 5 min, after which the supernatant was discarded. The pellet was resuspended in Dulbecco’s modified Eagle medium F12 (Hyclone, South Logan, UT, USA) with 10% fetal bovine serum (ABW, Uruguay) and 1% antibiotic mixture (penicillin/streptomycin, Invitrogen, USA). Finally, cells were plated at a density of 1 × 105 cells/ml in 6-well plates and incubated in a humidified atmosphere of 5% CO_2_ at 37°C. The media were changed every 2 or 3 days. Cells were passaged at 80% to 90% confluence using 0.25% trypsin-EDTA solution (passage 0). One-half of the articular chondrocytes at passage 0 were preserved at −80°C; the remainder were cultured and passaged for subsequent studies. Only passages 1 to 5 were used in the present study to avoid phenotype loss. In a cell viability experiment, the potential toxicity of CA (Sigma-Aldrich, St Louis, MO, USA) on chondrocytes was assessed using a commercially available CCK-8 assay kit (Dojindo Molecular Technologies Inc, Tokyo, Japan) according to the manufacturer’s instructions.

### Cell Viability

Cell viability was evaluated using the CCK-8 assay in accordance with the manufacturer’s instructions. Human OA chondrocytes were cultured in 96-well plates at a density of 8 × 10^3^ cells per well for 24 h and then pretreated with different concentrations of CA (0, 5, 10, 20, 50, 100, and 200 μmol/L) for an additional 24 h. The next day, the supernatant of each group was aspirated, and then washed with PBS, a medium containing 10-μL CCK-8 was added to each well and incubated at 37°C for 3 h. The optical density of each well was read at a wavelength of 450 nm by using a microplate reader (Leica Microsystems, Mannheim, Germany). Chondrocytes treated with dimethyl sulfoxide in the CCK-8 and subsequent *in vitro* experiments were used as the control group.

### NF-κB Overexpression

Human OA chondrocytes were transiently transfected with a NF-κB p65 plasmid or control vector (TSINGKE, BeiJing, China) for 24 h by using Lipofectamine 2000 (Invitrogen, USA) in accordance with the manufacturer’s instructions. Chondrocytes were pretreated with 20- and 50-μM CA for 24 h and then stimulated with LPS (10 μg/ml, Bioruler, Danbury, CT, USA) for 24 h. Messenger RNA (mRNA) and protein were collected for subsequent experiments.

### Real-Time Quantitative Polymerase Chain Reaction Analysis

Total RNA was extracted from chondrocytes by using the TRIzol reagent in accordance with the manufacturer’s instructions. Concentration was determined at 260 nm by using an ultraviolet spectrophotometer (NanoDrop 2000, Thermo-Fisher Scientific, Waltham, MA, USA), and the 260/280 ratio was calculated to verify quality and purity. Complementary DNA was generated by using a commercially available kit (PrimeScript Reverse Transcription kit, Takara Bio Inc, Kusatsu, Shiga Prefecture, Japan) under the following conditions: 15 min at 50°C and 5 sec at 85°C. Real-time quantitative polymerase chain reaction (RT-qPCR) was performed using a thermocycler (Mx3000P; Stratagene, La Jolla, CA, USA) and SYBR Premix Ex Taq II Kit (Takara Bio Inc, Japan) under the following conditions: 10 min at 95°C, followed by 40 cycles at 95°C for 15 s, and at 60°C for 1 min. Each gene analysis was performed in triplicate in accordance with the manufacturer’s recommendations. The RT-qPCR primers are listed in **Table 1**. Data were analyzed using the 2-ΔΔCT method.

**Table 1:** Reverse transcription-quantitative polymerase chain reaction primers.

Human-IL6-F	ACTCACCTCTTCAGAACGAATTG
Human-IL6-R	CCATCTTTGGAAGGTTCAGGTTG
Human-IL-1β-F	CTGTCCTGCGTGTTGAAAGA
Human-IL-1β-R	TTGGGTAATTTTTGGGATCTACA
Human-TNF-a-F	CCTCTCTCTAATCAGCCCTCTG
Human-TNFa-R	GAGGACCTGGGAGTAGATGAG
Human-MMP13-F	TCCTGATGTGGGTGAATACAATG
Human-MMP13-R	GCCATCGTGAAGTCTGGTAAAAT
Human-COX2-F	CTGGCGCTCAGCCATACAG
Human-COX2-R	CGCACTTATACTGGTCAAATCCC
Human-ADAMTS5- F	GAACATCGACCAACTCTACTCCG
Human-ADAMTS5-R	CAATGCCCACCGAACCATCT
Human-actin-F	GATTCCTATGTGGGCGACGA
human-actin-R	AGGTCTCAAACATGATCTGGGT

### Western Blot Analysis

Proteins were harvested from chondrocytes by using radioimmunoprecipitation assay (RIPA) and phenylmethylsulfonyl fluoride (PMSF) buffer (Beyotime Biotechnology, Shanghai, China), and 0.2-ml buffer was added in each group, left at 4°C for 20 min and centrifuged at 12000 rpm for 15 min. Then, the supernatant was discarded to determine the protein concentration in each sample by using a commercially available BCA protein assay kit (Thermo-Fisher Scientific, USA). A loading buffer was then added, mixed well, exposed to a metal bath at 95°C for 5 min, and stored at −80°C after dispensing. Western blot (WB) analysis was performed in accordance with a method described previously (Chen et al., 2019; Chen et al., 2020). Briefly, equal amounts of protein (40 μg) were separated using sodium-dodecyl polyacrylamide gel electrophoresis, loaded onto the 8%–12% separation gel according to the target molecular weight, and then transferred onto a polyvinylidene fluoride (PVDF) membrane. The PVDF membranes were incubated with a blocking buffer (5% nonfat milk in TBST), labeled with primary antibodies (Abcam, Cambridge, MA, USA) overnight at 4°C, washed 3 times with TBST, and incubated with horseradish peroxidase-labeled secondary antibodies (Abcam, USA). Primary antibodies against IL-1β, TNF-α, IL-6, and MMP-13 were purchased from Cell Signaling Technology (Cambridge, MA, USA); ADAMTS-5, p65, and IKB-α were purchased from Abcam (Cambridge). Images were developed after reaction with a high-sensitivity chemiluminescence reagent (Peprotech, London, United Kingdom).

### Enzyme-Linked Immunosorbent Assay

Briefly, chondrocyte supernatants were collected by centrifugation at 176 g for 5 min. The proteins of the chondrocyte supernatants in each group were also extracted with RIPA and PMSF buffer, and then quantified with the BCA kit. Cytokine concentrations (IL 1β, IL 6, and TNF-α) were determined using a commercially available enzyme-linked immunosorbent assay (ELISA) kit in accordance with the manufacturer’s instructions (Proteintech Group Inc, Rosemont, IL, USA).

### Statistical Analyses

All the experiments were performed at least 3 times independently, and data are reported as mean ± standard deviation. All the analyses were performed using SPSS version 23.0 (IBM Corporation, Armonk, NY, USA). Differences between groups were compared using one-way analysis of variance, and a p values of <0.05 was considered statistically significant.

## Results

### Cytotoxicity Assay: Effect of CA on Human OA Chondrocyte Viability

The potential cytotoxicity of different concentrations of CA (0, 5, 10, 20, 50, 100, and 200 μM) on human OA chondrocytes was examined using the CCK-8 assay. The results suggested that low concentrations of CA (10, 20, and 50 μM) promoted chondrocyte viability, while high concentrations (100 and 200 μM) of CA could reversed it (**Figure 1B**). Therefore, CA at concentrations of 10, 20, 50 μmol/L were used in subsequent experiments.

### CA Inhibited the Expression of Proinflammatory Cytokines in LPS-Induced Human OA Chondrocytes

**Figure 2:**
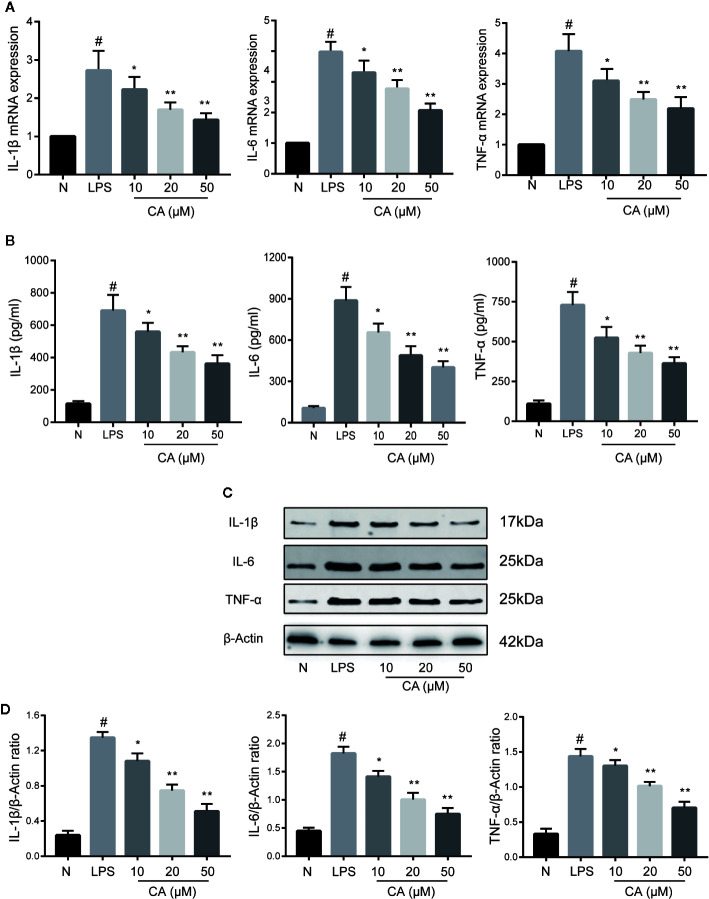
Effect of cinnamic aldehyde (CA) on lipopolysaccharide (LPS)-induced interleukin (IL)-1β, IL-6, and tumor necrosis factor-alfa (TNF-α) expression in human osteoarthritis (OA) chondrocytes. Human OA chondrocytes were pretreated for 24 h with various concentrations of CA (10, 20, and 50 μM) and then stimulated or not stimulated with LPS (10 μg/ml) for 24 h. **(A)** Messenger RNA (mRNA) expression levels of IL-1β, IL-6, and TNF-α were determined using real-time quantitative polymerase chain reaction (RT-qPCR). **(B)** Enzyme-linked immunosorbent assay results for IL-1β, IL-6, and TNF-α in chondrocyte supernatants. **(C, D)** Protein expression levels were assayed by Western blot and quantification analysis using Image J software. ^#^P < 0.01 compared with control group; *P < 0.05, **P < 0.01 compared with LPS group. All experiments were performed at least 3 times independently ( n = 3).

The effects of CA on proinflammatory cytokines (IL-1β, IL-6, and TNF-α) on LPS-induced human OA chondrocytes were investigated using RT-qPCR, WB, and ELISA. As shown in **Figure 2**, compared with the normal group, LPS-induced chondrocytes exhibited significantly increased mRNA and protein expression levels of IL-1β, IL-6, and TNF-α (p < 0.05). However, pretreatment with different concentrations of CA (10, 20, and 50 μM) significantly downregulated the mRNA and protein expression levels of IL-1β, IL-6, and TNF-α in a dose-dependent manner (p < 0.05).

### CA Inhibited the Expressions of MMP13 and ADAMTS-5 in LPS-Induced Human OA Chondrocytes

**Figure 3:**
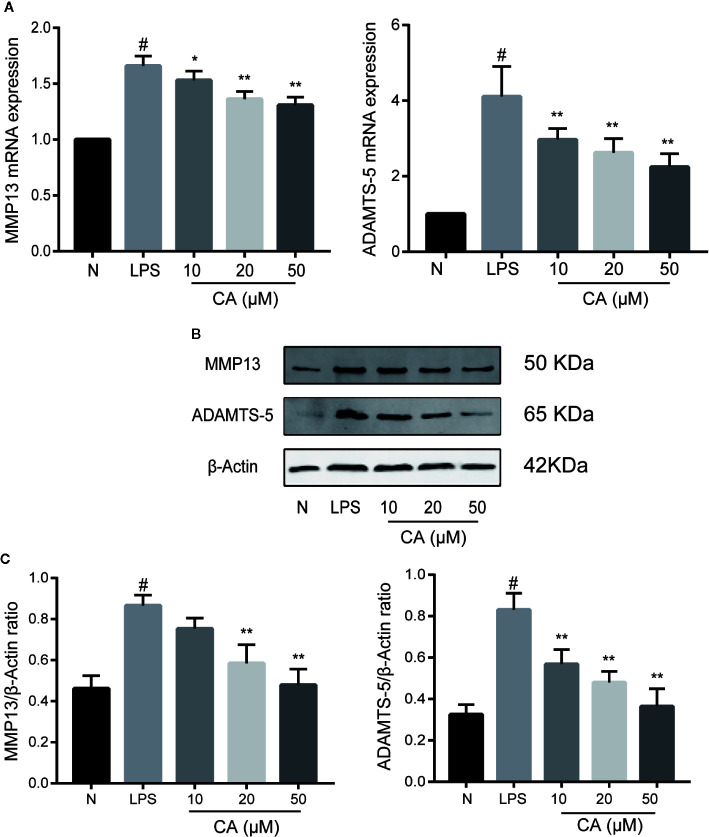
Effect of cinnamic aldehyde (CA) on lipopolysaccharide (LPS)-induced matrix metalloproteinase (MMP)13 and a disintegrin and metalloproteinase with thrombospondin motifs 5 (ADAMTS-5) expression in human osteoarthritis (OA) chondrocytes. Human OA chondrocytes were pretreated for 24 h with various concentrations of CA (10, 20, and 50 μM) and then stimulated or not stimulated with LPS (10 μg/ml) for 24 h. **(A)** Messenger (mRNA) expression levels of MMP13 and ADAMTS-5 were determined using real-time quantitative polymerase chain reaction (RT-qPCR). **(B, C)** Protein expression levels were assayed using Western blot and quantification analysis with Image J software. ^#^P < 0.01 compared with control group; *P < 0.05, **P < 0.01 compared with LPS group. All experiments were performed at least 3 times independently (n = 3).

Subsequently, the effects of CA on the mRNA and protein levels of MMP13 and ADAMTS-5 were assessed. As presented in **Figure 3A**, LPS-induced chondrocytes exhibited markedly upregulated mRNA expression levels of MMP13 and ADAMTS-5 (p < 0.05), whereas the expression levels of MMP13 and ADAMTS-5 were significantly reduced in the CA pretreated group in a dose-dependent manner (p < 0.05). Similarly, WB analysis demonstrated that CA decreased the protein upregulation of MMP13 and ADAMTS-5 (p < 0.05; **Figures 3B, C**). Therefore, 20- and 50-μM CA were used in the subsequent experiments.

### CA Inhibited Activation of the NF-κB Pathway in LPS-Induced Human OA Chondrocytes

**Figure 4:**
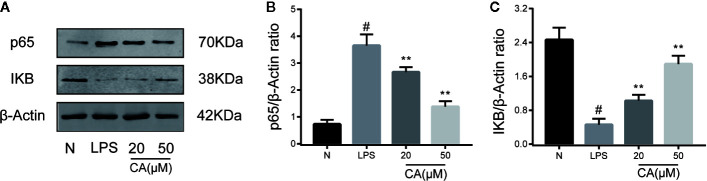
Effect of cinnamic aldehyde (CA) on lipopolysaccharide (LPS)-induced nuclear factor kappa-light-chain-enhancer of activated B cells (NF-κB) activation in human osteoarthritis (OA) chondrocytes. Human OA chondrocytes were pretreated for 24 h with different concentrations of CA (20, 50 μM) and then stimulated or not stimulated with LPS (10 μg/ml) for 24 h. The protein expression levels of p65, inhibitory protein of NF-κB (IKB-α) were determined using Western blot assay **(A)** and then quantified **(B, C)**. ^#^P < 0.01 compared with control group; **P < 0.01 compared with LPS group. All experiments were performed at least 3 times independently (n = 3).

To further investigate the potential anti-inflammatory mechanism of CA, biomarkers of the NF-κB pathway (p65 and IKB-α) were detected using a WB assay. As presented in **Figure 4**, LPS induced significant upregulation and expression of p65 in human OA chondrocytes (p < 0.05). By contrast, pretreatment with various concentrations of CA (20 and 50 μM) significantly decreased the expression level of p65. In addition, stimulation of chondrocytes with LPS significantly inhibited the expression of IKB, while pretreatment with CA (20 and 50 μM) significantly promoted the expression of IKB (p < 0.01).

### CA Reversed Inflammation *via* the NF-κB Pathway

**Figure 5:**
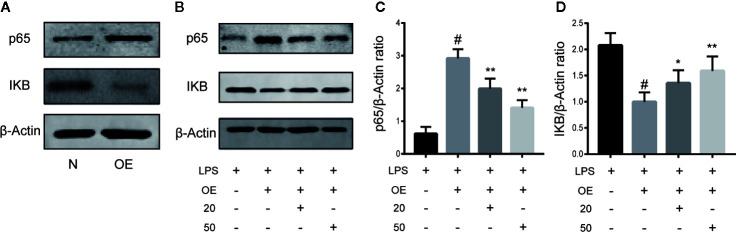
Cinnamic aldehyde (CA) reverse inflammation *via* the nuclear factor kappa-light-chain-enhancer of activated B cells (NF-κB) pathway. Human osteoarthritis (OA) chondrocytes were transiently transfected with a NF-kB p65 plasmid or an empty vector for 24 h using Lipofectamine 2000 (Invitrogen, USA). **(A)** Chondrocytes were pretreated with 20 μM and 50 μM CA for 24 h and then stimulated with lipopolysaccharide (LPS) for 24 h. **(B–D)** Treatment with an NF-κB p65 plasmid significantly activated the NF-κB signal pathway. Pretreatment with CA rescued the expression of p65 and inhibitory protein of NF-κB (IKB-α). OE, overexpression; 20, 20 μM CA; 50, 50 μM CA. ^#^P < 0.01 compared with LPS group; *P < 0.05, **P < 0.01 compared with LPS+OE group. All experiments were performed at least 3 times independently (n = 3).

To demonstrate the necessity of the NF-κB pathway in CA to reduce cartilage damage and inhibit inflammation, chondrocytes were transiently transfected with NF-κB p65 plasmid or control vector for 24 h. As presented in **Figure 5A**, stimulation of chondrocytes with the NF-κB p65 plasmid significantly activated the NF-κB signal pathway. As is shown in **Figures B–D**, after induction with LPS, the p65 expression level was significantly upregulated and the IKB expression level downregulated in the LPS + overexpression (OE) group, while CA rescued the p65 and IKB expressions (p < 0.05).

## Discussion

Cartilage degeneration is one of the main pathological manifestations of OA. The results of this study indicate that pretreatment with CA significantly inhibited the productions of IL-1β, IL-6, and TNF-α induced by LPS in human OA chondrocytes, and significantly reduced the expression levels of MMP-13 and ADAMTS-5. Furthermore, pretreatment with CA inhibited the expressions of NF-κB, p65, and IKB-α induced by LPS in chondrocytes. Pretreatment with CA significantly reversed the expressions of p65 and IKB-α in LPS-induced human OA chondrocytes when transfected with a NF-κB p65 plasmid.

Cinnamon, a common traditional Chinese medicine, is widely used in the treatment of low back pain, diabetes, arthritis, and cardiovascular diseases. CA is an active ingredient extracted from cinnamon, and its anti-inflammatory property is one of its most important characteristics. Cinnamon extract can inhibit the production of prostaglandins by inhibiting COX, thereby inhibiting inflammation (Butt et al., 2013). CA exerts anti-inflammatory and antioxidative effects in an LPS-induced mouse macrophage cell line (Kim et al., 2010). In the LPS-induced RAW 264.7 cell line, trans-cinnamaldehyde decreased the transcriptional activity of NF-κB by inhibiting its DNA binding activity (Reddy et al., 2004). CA can also inhibit *Aggregatibacter actinomycetemcomitans*-induced inflammation by activating autophagy (Chung et al., 2018). In addition, CA can modulate nuclear factor erythroid 2-related factor 2 (Nrf2) and then antioxidative stress (Kim et al., 2017; Abou El-Ezz et al., 2018). Reactive oxygen species (ROS) can activate the NF-KB signaling pathway and release more inflammatory factors (Kizaki et al., 2002), and the property of the activated NF-KB signaling pathway induced by CA can be interrupted by dithiothreitol (Macpherson et al., 2007; Kim et al., 2010). However, to our knowledge, no published studies have investigated the therapeutic mechanism of CA in OA. In this study, we validated the effect of CA on chondrocyte inflammation and degeneration *in vivo* and found that CA can significantly inhibit the expressions of inflammatory factors and reduce the loss of ECM.

Inflammatory cytokines play important roles in promoting the pathogenesis of OA. Among the inflammatory cytokines, the interleukin family, especially IL-1β and IL-6, has been considered to play a critical role in the pathological development of OA (Santangelo et al., 2012; Laavola et al., 2018). It can stimulate the productions of the inflammatory cytokines NO, iNOS, and prostaglandin E2 (PGE2), which are associated with OA progression (Liu-Bryan, 2013; Suantawee et al., 2015). TNF-α is another inflammatory cytokine that drives the pathogenesis and progression of OA (Larsson et al., 2015). TNF-α can stimulate the expressions of MMPs, ADAMTS, and chondrocyte dedifferentiation markers (Murakami et al., 2000; Shi et al., 2004). MMP13, a member of the MMPs family of enzymes that cleave protein substrates based on a conserved mechanism, promotes cartilage turnover and breakdown, degrades components of the ECM, and plays an important pathological role during inflammation in OA (Abeles and Pillinger, 2006; Klein and Bischoff, 2011; Zheng et al., 2017). ADAMTS consists of secreted zinc MMPs with thrombospondin motifs, another family with an important role in the breakdown type II collagen and proteoglycans that constitute the main structure of the ECM (Verma and Dalal, 2011; Malemud, 2019). ADAMTS-5 is a member of the ADAMTS family and plays a primary role in degradation of ECM in OA (Verma and Dalal, 2011; Larkin et al., 2015). Therefore, inhibiting the expressions of proinflammatory cytokines (IL-1β, IL-6, and TNF-α) and reducing the loss of collagen-II and ECM may have a significant mitigating effect on the progression of OA.

To further investigate the potential anti-inflammatory and chondroprotective mechanisms of CA, we examined the expressions of the NF-κB signaling pathway biomarkers in pretreatment with or without CA in LPS-induced human OA chondrocytes. The NF-κB pathway is one of the major catabolic pathways involved in the pathogenesis of OA and plays a key role in the regulation of inflammatory mediators associated with OA (Rigoglou and Papavassiliou, 2013). Upon stimulation, the activated NF-κB pathway regulated the expressions of an array of cytokines that induce ECM destruction, aggravating the OA process (Marcu et al., 2010). Therefore, inhibition of the NF-κB signaling pathway activity may be a potential therapeutic target for OA. The results of the present study demonstrated that CA significantly inhibited the NF-κB signaling pathway activity in LPS-induced human OA chondrocytes. Moreover, we found that CA specifically inhibited the activity of the NF-kB signaling pathway when transfected with a NF-κB p65 plasmid in human OA chondrocytes, which is consistent with the results of several previous studies (Reddy et al., 2004; Kim et al., 2007; Marcu et al., 2010).

Collectively, the results of this study were the first to demonstrate the anti-inflammatory and chondroprotective effects of CA in an *in vivo* model. In LPS-induced human OA chondrocytes, CA significantly decreased the expression levels of IL-1β, IL-6, TNF-α, MMP13, and ADAMTS-5 by inhibiting the NF-κB signaling pathway. As such, CA may be an ideal therapeutic agent for treating OA.

## Data Availability Statement

All datasets generated for this study are included in the article/supplementary material.

## Ethics Statement

This study was approved by the Medical Ethics Committee of Beijing University of Chinese Medicine Third Affiliated Hospital (BZYSY-2019KYKTPJ-26).

## Author ContributionS

Article design: PC, QW. Article performance: PC, AR, JZ, XZ, YM. Data extraction: JZ, XZ. Article writing, PC, LH.

## Funding

This article was founded by General Program of National Natural Science Foundation of China (No.81373662), Capacity building project of Chinese and western medicine clinical collaboration on major difficult disease in 2019, and General Program of National Natural Science Foundation of China (No.81874475).

## Conflict of Interest

The authors declare that the research was conducted in the absence of any commercial or financial relationships that could be construed as a potential conflict of interest.
